# Schwann Cell-Derived Exosomes Induce the Differentiation of Human Adipose-Derived Stem Cells Into Schwann Cells

**DOI:** 10.3389/fmolb.2021.835135

**Published:** 2022-01-31

**Authors:** Nan Zhou, Zhao Xu, Xiang Li, Sen Ren, Jing Chen, Hewei Xiong, Cheng Wang, Jiahe Guo, Yu Kang, Zhenbing Chen, Wenqing Li, Xiaofan Yang, Xing Zhang, Xiang Xu

**Affiliations:** ^1^ Department of Hand Surgery, Union Hospital, Tongji Medical College, Huazhong University of Science and Technology, Wuhan, China; ^2^ Department of Orthopedics, Trauma and Reconstructive Surgery, RWTH Aachen University Hospital, Aachen, Germany; ^3^ Department of Clinical Laboratory, Huai’an Second People’s Hospital, The Affiliated Huai’an Hospital of Xuzhou Medical University, Huai’an, China; ^4^ Department of Hand and Foot Surgery, Huazhong University of Science and Technology Union Shenzhen Hospital, Shenzhen, China

**Keywords:** adipose-derived stem cell, exosome, Schwann cell, differentiation, miRNA sequencing

## Abstract

Adipose-derived stem cells (ADSCs) can differentiate into Schwann cells (SCs) at the site of nerve injury, where Schwann cell-derived exosomes (SC-Exos) are suspected to exert an induction effect. Our study aimed to induce the differentiation of ADSCs *in vitro* using SC-Exos and to investigate the mechanisms involved through miRNA sequencing. Subcutaneous fat was used to extract ADSCs. Exosomes were extracted from Schwann cell lines (RSC96) using ultracentrifugation and were able to be taken up by human ADSCs. After 8 days of induction of ADSCs by SC-Exos, phenotypic characteristics were observed by examining the expression of SC markers (S100ß, NGFR, MPZ, GFAP) through RT-qPCR, Western blot and immunofluorescence. The RNA and protein expression levels of S100ß, NGFR, MPZ, and GFAP were found to be significantly higher in the SC-Exo induction group than in the uninduced group, which was also consistent with the immunofluorescence results. Additionally, miRNA sequencing was performed on exosome-induced ADSCs, followed by bioinformatic analysis and validation of the results. According to the sequencing results, there were a total of 94 differentially expressed miRNAs. Bioinformatics analysis indicated that 3506 Gene Ontology terms and 98 Kyoto Encyclopedia of Genes and Genomes pathways were significantly enriched. Ten miRNAs, 5 target mRNAs and elevated expression of the PIK3CD/Akt pathway were validated by RT-qPCR or Western blot, which is consistent with the sequencing results. Our study demonstrates that the utility of SC-Exos is effective in inducing the differentiation of ADSCs into SCs, in which these validated differentially expressed miRNAs exert a vital effect. This work provides a new paradigm *via* rationally applying Schwann cell-derived exosomes as a promising therapeutic option for repairing peripheral nerve injury.

## Introduction

Peripheral nerve injury (PNI) is one of the common complications of various types of trauma and mainly occurs after motor vehicle accidents, natural disasters, work-related accidents and sports injuries (Wang et al., 2019). The consequences of peripheral nerve injury are often devastating, resulting in varying degrees of sensory and motor impairment and long-term inconvenience to the patient’s life. Spontaneous peripheral nerve repair is strictly limited by the type and extent of injury, and functional recovery is limited due to inflammation, scar formation, and the wrong regeneration direction of sensory and motor nerves (Stoll et al., 2002). For nerve injury with complete gap transection, autologous nerve transplantation is its main treatment, but it also has disadvantages such as limited graft length, denervation of the donor site and the need for multiple surgeries (Dahlin, 2008). There is therefore an urgent need to find a new treatment to treat peripheral nerve injury.

At present, cell therapy is considered to be a promising method in the field of nerve repair, especially the transplantation of mesenchymal stem cells (MSCs). Many *in vivo* experiments in animals have shown that transplantation of MSCs can promote injured peripheral nerve regeneration. However, the possible mechanisms have been confirmed by subsequent studies to exist in two aspects: transplanted MSCs can secrete various types of neurotrophic factors, such as brain-derived neurotrophic factor (BDNF), nerve growth factor (NGF), glial growth-like factor (GGF), and neuregulin-1 (NRG-1), to promote nerve repair (Jiang et al., 2017; Lavorato et al., 2021); transplanted MSCs are stimulated to differentiate into Schwann cells (SCs) in the nerve injury area *in vivo* (Chen et al., 2006; Ladak et al., 2011), and Schwann cells can provide growth channels for axonal regeneration by forming Büngner bands, so that axons can continue to grow across the defect area. However, the specific reasons and mechanisms for inducing MSCs to differentiate into Schwann cells are still unclear.

Exosomes are a class of microvesicles with phospholipid bilayers that are found and abundantly present in human body fluids (e.g., blood, saliva, ascites, cerebrospinal fluid) (Hessvik and Llorente, 2018); they begin with endosomes formed by internalization of the cytoplasmic membrane and are secreted into the extracellular space after maturation to mediate intercellular communication and exert physiological functions by transferring mRNAs, lncRNAs, miRNAs, and proteins (Zhang et al., 2015). After peripheral nerve injury, Wallerian degeneration occurs at the distal end of the nerve, and Schwann cells show proliferation and dedifferentiation. It has been reported that dedifferentiated Schwann cells secrete large amounts of exosomes into the surrounding microenvironment after nerve injury and participate in the repair of the injured nerve (Lopez-Leal and Court, 2016). In addition, *in vitro* experiments have confirmed that MSCs cocultured with Schwann cells differentiate into Schwann cells (Wei et al., 2010; Zhu et al., 2015), indicating that a paracrine substance of Schwann cells induces MSC differentiation. These studies suggest that the differentiation of transplanted MSCs into Schwann cells may be accomplished by Schwann cell exosome induction. In this study, we aimed to investigate the effect of Schwann cell-derived exosomes on inducing the differentiation of human adipose-derived stem cells (ADSCs) into Schwann cells *in vitro* and explored the possible mechanism by sequencing the miRNAs in the induced cells.

## Methods and Materials

### ADSCs Isolation and Culture

Human subcutaneous fat tissue was harvested from healthy donors who underwent skin flap surgery, which was reviewed and approved by the Ethics Committee of Tongji Medical College, Huazhong University of Science and Technology (No. 2018-S288). Written informed consent was obtained from each donor before donation. ADSCs were isolated and cultured according to our laboratory’s standardized protocols (Ren et al., 2019).

### ADSCs Characterization

Flow cytometry was used to observe the ADSC phenotypes. Passage 3 ADSCs were trypsinized and incubated with anti-CD34-PE, anti-CD44-PE, anti-CD73-FITC, anti-CD90-FITC, and anti-HLA-DR-PE (Biolegend, San Diego, CA, USA). After three washes with PBS, the fluorescence of ADSCs was observed. To verify adipogenic differentiation and osteogenic differentiation, ADSCs at passage 3 were incubated separately with adipogenic and osteogenic differentiation medium (Gibco, United States) following the supplier’s instructions for 2 or 3 weeks. The induced cells were stained with Oil Red O and Alizarin Red respectively and microscopically imaged.

### RSC96-Derived Exosomes Isolation and Identification

Exosomes were isolated from the supernatant of a rat Schwann cell line (RSC96) using a previous protocol (Cosenza et al., 2018). When RSC96s reached 80–90% confluence, they were grown in exosome-free culture medium, which consisted of 10% exosome-free FBS (Serapro, USA) obtained by ultracentrifugation at 120,000 g for 12 h. The supernatants were collected and initially centrifuged at 1000 g for 10 min to remove dead cells and later centrifuged at 4000 g for 30 min to remove cell debris. Subsequently, the supernatants were centrifuged at 120,000 g for 70 min and 120,000 g for 60 min at 4°C. The pellets were resuspended in PBS and stored at −80°C.

The morphology of RSC96-derived exosomes (RSC96-Exos) was observed under a transmission electron microscope (TEM; Hitachi, Japan). Nanoparticle tracking analysis (NTA; Beckman Coulter, United States) was used to measure the size distribution of RSC96-Exos. The total protein concentration was quantified with a Pierce BCA Protein Assay Kit (Aspen, China) according to the manufacturer’s instructions.

### RSC96-Exos Labeling and Uptake Assay

RSC96-Exos were incubated with red fluorescent dye (PKH26, Sigma, United States) for 5 min and treated with 0.5% BSA/PBS to neutralize redundant dye. Then, the labeled exosomes were centrifuged to remove contaminating dye. For the uptake assay, ADSCs at the proper density were incubated with PKH26-labeled RSC96-Exos (20 μg/ml) in a 35 mm confocal dish. After incubation for 24 h, the ADSCs treated with RSC96-Exos were signed with FITC phalloidin (Yeasen Biotech Co., Shanghai, China) and DAPI (Solarbio, Beijing, China) before image acquisition. Exosome uptake by ADSCs was observed by laser scanning confocal microscope.

### Differentiation of ADSCs Into Schwann Cells

The induction of ADSCs was performed by two methods. Briefly, when ADSCs were induced by Dezawa’s method (In-ADSCs) (Dezawa et al., 2001), ADSCs were incubated with a-MEM (Gibco; Thermo Fisher Scientific, Inc.) containing 1 mM b-mercaptoethanol (b-ME; Sigma-Aldrich, St. Louis, MO) for 24 h and then with a-MEM supplemented with 10% FBS (Gibco; Thermo Fisher Scientific, Inc.) and 35 ng/ml all-trans retinoic acid (ATRA; Sigma-Aldrich) for 3 days, followed by incubation for 4 days in original differentiation medium consisting of a-MEM, 10% FBS, and a mixture of 5 mM forskolin (FSK; Sigma-Aldrich), 10 ng/ml human basic fibroblast growth factor (b-FGF; PeproTech, Inc., Rocky Hill, NJ), 5 ng/ml human platelet-derived growth factor (PDGF; PeproTech), and 200 ng/ml human heregulin-b1 (HRG-b1; PeproTech). On the other hand, ADSCs induced by RSC96-Exos (Exo-ADSCs) were cultured a-MEM supplemented with 10% FBS and RSC96-Exos (20 μg/ml) for 8 days, and the media was changed every 2 days with the exosomes replenished each time. In the control group, ADSCs were cultured in α-MEM supplemented with 10% FBS. Fully induced ADSCs were used in the subsequent *in vitro* experiments.

### RT-qPCR

RT-qPCR was performed in accordance with a previously published method (Ren et al., 2021). Total RNA was isolated with the miRNeasy Mini Kit (Qiagen, Germany) according to the manufacturer’s instructions. MRNAs and miRNAs were reversely transcribed into cDNA using the Prime-Script® RT reagent Kit (#RR047A, TaKaRa) and Mir-X™ miRNA First-Strand Synthesis kit (#RR638315, TaKaRa), respectively. Real-time PCR was performed on the StepOnePlus™ platform (Applied Biosystems, USA) using TB Green® Premix Ex Taq™ II kit (#RR820A, TaKaRa). The primer sequences are presented in Supplementary Tables S1, S2. The relative expression levels of targeted genes were calculated using the 2^−ΔΔCt^ method and normalized to GAPDH or U6.

### Western Blotting

Western blotting was performed in accordance with a previously published method (Chen et al., 2019). Total proteins were extracted by RIPA lysis buffer with proteinase inhibitor (Roche, Switzerland). In total, 30 μg aliquots of protein were separated in 10% SDS-PAGE gels and then transferred onto PVDF membranes (Millipore, United States). After 1.5 h of blocking with 5% w/v nonfat dry milk buffer, the membrane was incubated overnight with primary antibodies against GM130 (#66662-1-Ig, Proteintech), GAPDH (#10494-1-AP, Proteintech), CD63 (#PA5-92370; Invitrogen), CD9 (#ab92726, Abcam), CD81 (#ab109201, Abcam), S100ß (#ab52642; Abcam), nerve growth factor receptor (NGFR, #8238, Cell Signaling Technology), myelin protein zero (MPZ, #ab31851, Abcam), glial fibrillary acidic protein (GFAP, #3670, Cell Signaling Technology), phosphatidylinositol-4,5-bisphosphate 3-kinase catalytic subunit delta (PIK3CD, #ab109006, Abcam) and phosphorylated serine/threonine kinase (p-AKT, #4060, Cell Signaling Technology). Then, the membrane was incubated with secondary antibodies (Aspen, China) and exposed to X-ray film (UVP, United States).

### Immunocytochemistry

Immunocytochemistry analysis was performed as previously described (Kang et al., 2019). To identify the phenotypic changes in differentiated ADSCs, the expression levels of SC markers were recognized by immunophenotype using primary antibodies specific for S100ß (1:100, Abcam), NGFR (1:100, Cell Signaling Technology), MPZ (1:100, Abcam) and GFAP (1:300, Cell Signaling Technology). Uninduced ADSCs were used as a negative control. The images were taken by fluorescence microscopy.

### MiRNA-Seq

Total RNA from Exo-ADSCs and uninduced ADSCs was isolated for microRNA analyses using the mirVana miRNA Isolation Kit (Ambion) according to the manufacturer’s protocol. Quantitation of total RNA was carried out using a Nanodrop 2000 (Thermo Fisher Scientific Inc., United States), while RNA integrity was assessed by an Agilent 2100 Bioanalyzer (Agilent Technology, United States). Standard cDNA libraries were constructed and sequenced using the Illumina NovaSeq6000 platform. Small RNA sequencing and analysis were conducted by OE Biotech Co., Ltd. (Shanghai, China). The sequence data were filtered to obtain clean data, and then aligned and subjected to the Bowtie (Langmead et al., 2009) search against Rfam v.10.1 (Griffiths-Jones et al., 2003). The known miRNAs were identified by alignment against the miRBase database (Griffiths-Jones et al., 2008), while unannotated reads were analyzed by mirdeep2 (Friedländer et al., 2012) to predict novel miRNAs. Based on the hairpin structure of a pre-miRNA and the miRBase database, the corresponding miRNA star sequence and miRNA mature sequence were also identified. The expression level of miRNAs was determined by the transcript per million (TPM). Furthermore, differential expression analysis was performed using DEGseq (Wang et al., 2010). Criteria applied in selected differentially expressed miRNAs were *p* < 0.05 and fold-change ≥ 2 or ≤0.5. Detailed RNA-seq data have been deposited at Gene Expression Omnibus (http://www.ncbi.nlm.nih.gov/geo; GSE183623).

### MiRNA Target Prediction, GO and KEGG Enrichment Analyses

The MiRanda database (http://www.microrna.org/microrna/home.do) was used to predict target genes of selected differentially expressed miRNAs. Gene Ontology (GO; http://geneontology.org) and Kyoto Encyclopedia of Genes and Genomes (KEGG; https://www.genome.jp/kegg/) pathway analyses were applied to determine the roles of miRNA target genes. We used the hypergeometric distribution test to identify significantly enriched gene sets. A *p* value <0.05 was considered statistically significant. MiRNA-mRNA interactions were selected to generate a network map with Cytoscape software (http://www.cytoscape.org/).

### Statistical Analysis

All data were expressed as the mean ± SEM. Comparisons between the two groups were evaluated with unpaired Student’s t test. For group ≥3, one-way ANOVA with Bonferroni post hoc test was employed. Statistical significance was set at *p* < 0.05. Statistical analysis was performed using GraphPad Prism v 7.0 software.

## Results

### Identification of ADSCs

**FIGURE 1:**
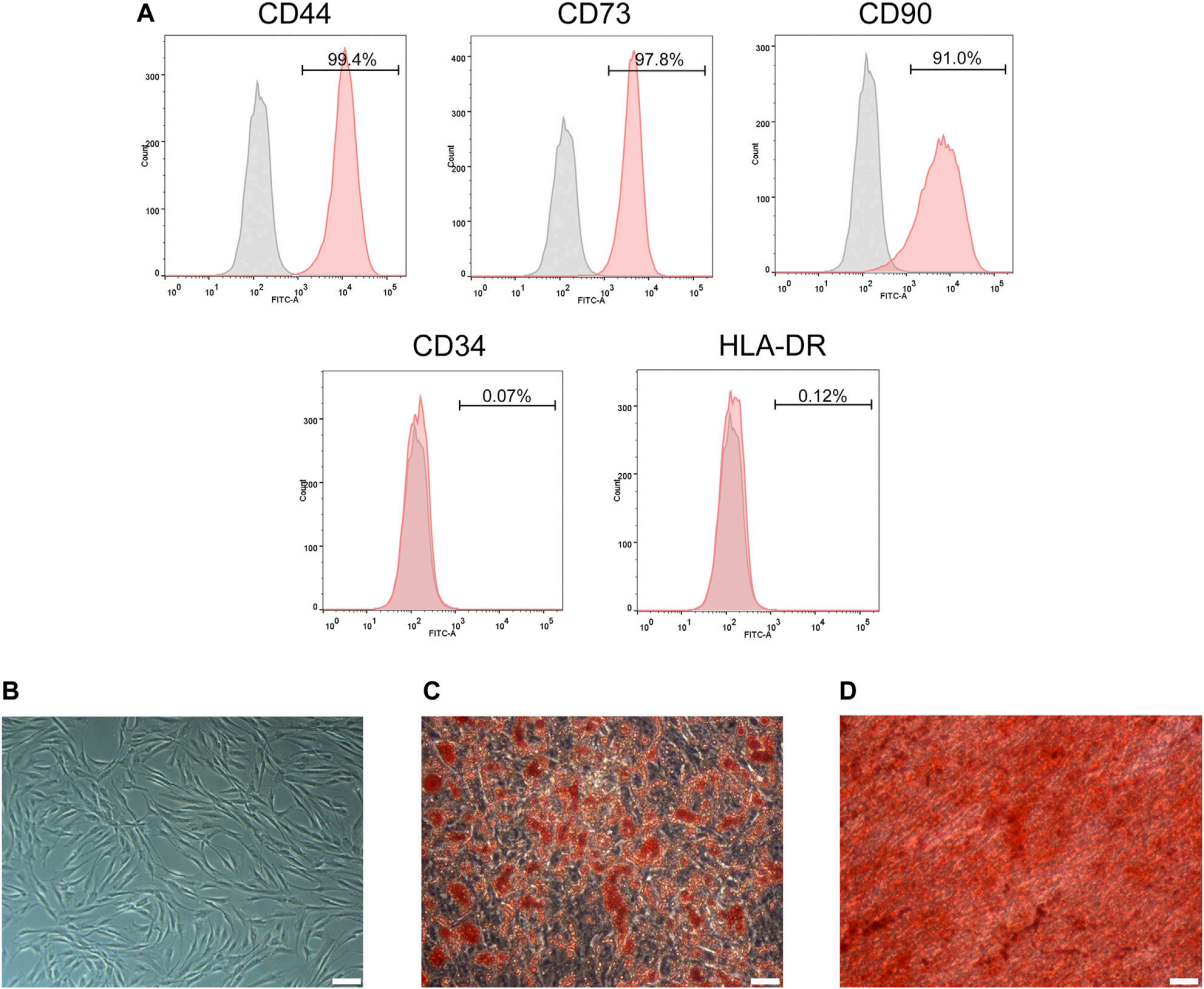
Identification of ADSCs. **(A)** Flow cytometry analysis showed that ADSCs were positive for CD44, CD73 and CD90 but negative for CD34 and HLA-DR. **(B)** The morphology of ADSCs. **(C)** Adipogenic potentials of ADSCs (oil red O staining). **(D)** Osteogenic potentials of ADSCs (alizarin red staining). **(A,C,D)** Scale bars: 100 mm.

ADSCs extracted from the subcutaneous fat of the skin flap were transmitted to the P3 generation and cultured for 3–5 days, and ADSCs demonstrated a typical flattened structure of fibroblast-like and shuttle-shaped morphology (Figure 1B). ADSCs were identified by flow cytometry for surface markers: CD44 (99.4%), CD73 (97.8%), and CD90 (91.0%) were positive, while CD34 (0.07%) and HLA-DR (0.12%) were negative (Figure 1A). In addition, adipogenesis and osteogenesis assays were verified by the formation of refractive lipid droplets after staining with Oil Red O (Figure 1C) and calcified nodules stained with Alizarin Red (Figure 1D). The above results indicate that we successfully extracted primary ADSCs with differentiation potential.

### Identification and Internalization of RSC96-Exos

**FIGURE 2:**
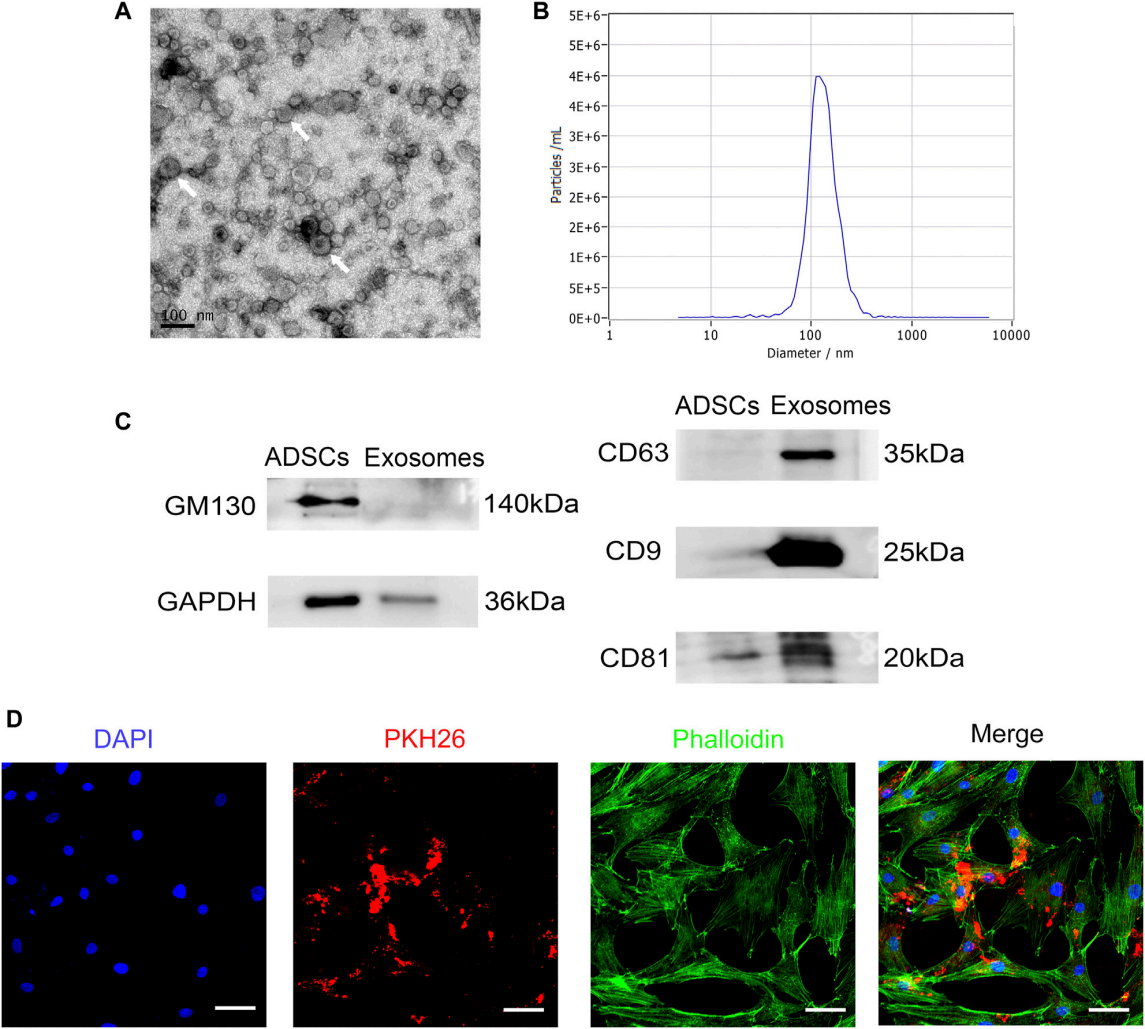
Identification and internalization of RSC96-Exos. **(A)** Morphology of RSC96-Exos observed by transmission electron microscopy. Scale bar: 100 nm. White arrows indicate the RSC96-Exos. **(B)** Measurement of the RSC96-Exos population by nanoparticle tracking analysis demonstrated a single-peaked pattern (127.5 ± 2.1 nm in diameter). **(C)** Western blot analysis of total protein markers (GM130 and GAPDH) and exosome surface markers (CD63, CD9 and CD81) in ADSCs and RSC96-Exos. **(D)** Confocal images of ADSCs incubated with 20 μg PKH26-labeled RSC96-Exos for 24 h. Scale bar: 50 µm.

RSC96-Exos showed a typical disc-like structure under TEM (Figure 2A). In addition, the average diameter of the extracted exosomes was determined to be 127.5 ± 2.1 nm by NTA (Figure 2B). Detection of exosomal markers by Western blot revealed that CD9, CD63, and CD81 were positive, while nonexosomal markers, such as GM130, were negatively expressed, and GAPDH were weakly expressed relative to ADSCs (Figure 2C). These results infer that RSC96-Exos were successfully isolated.

To determine whether RSC96-Exos are internalized by ADSCs, ADSCs were incubated with 20 μg/ml PKH26-labeled RSC96-Exos for 24 h. The results showed that red fluorescent signals were obviously detected in ADSCs, indicating that RSC96-Exos were taken up by ADSCs (Figure 2D).

### Phenotypic Characteristics of In-ADSCs and Exo-ADSCs

**FIGURE 3:**
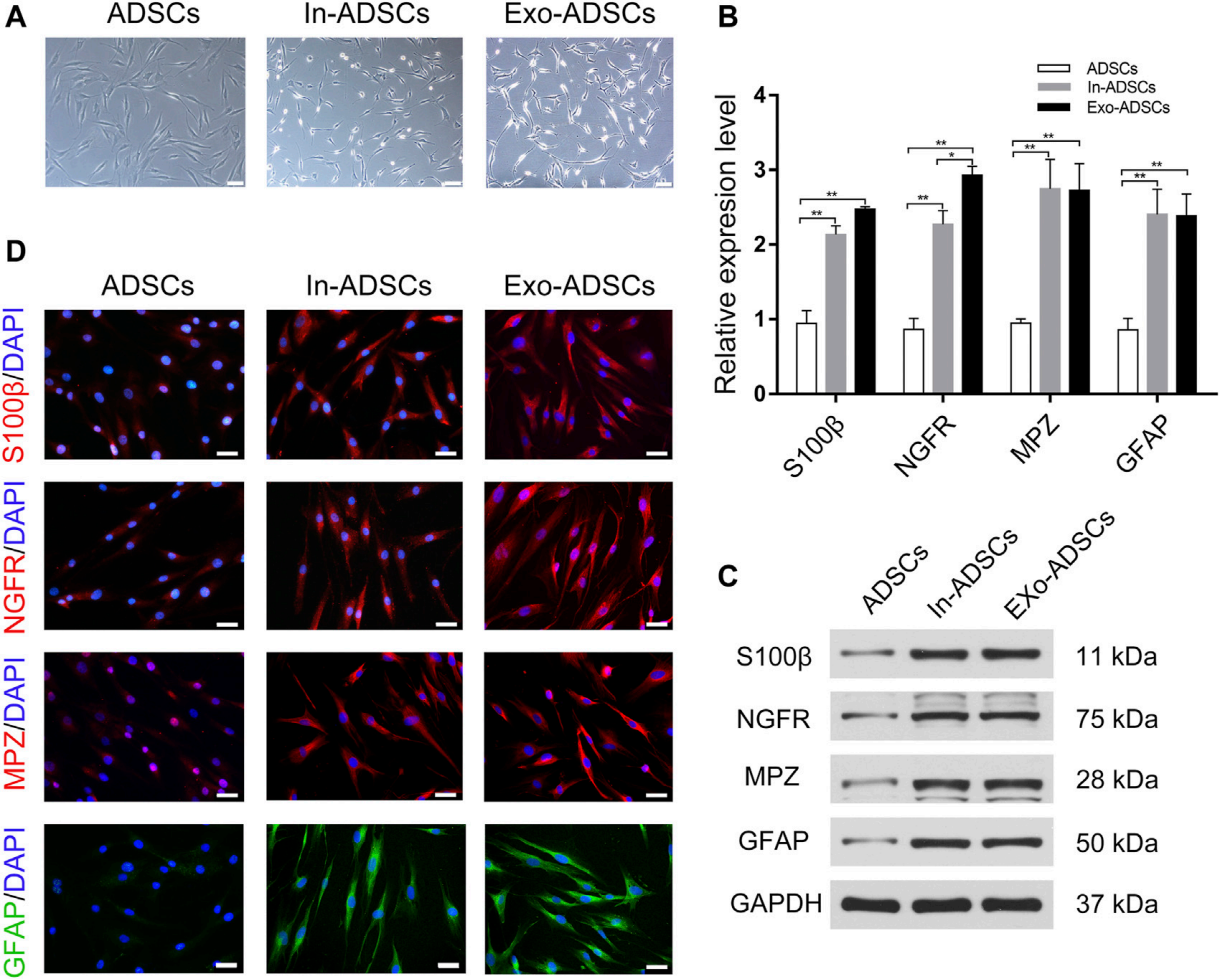
Phenotypic characteristics of In-ADSCs and Exo-ADSCs. **(A)** Images of ADSCs, In-ADSCs, and Exo-ADSCs taken under a phase-contrast microscope. Scale bar: 100 µm. **(B)** RT-qPCR analysis showing the mRNA levels of S100ß, NGFR, MPZ and GFAP in ADSCs, In-ADSCs, and Exo-ADSCs. **(C)** Western blot analysis showing the protein levels of S100ß, NGFR, MPZ and GFAP in ADSCs, In-ADSCs, and Exo-ADSCs. **(D)** Fluorescence microscopy showing the immunoreactivity of S100ß, NGFR, MPZ and GFAP in ADSCs, In-ADSCs, and Exo-ADSCs. Scale bar: 50 µm. The data was presented as the mean ± SD (*n* = 3 independent experiments). *p < 0.05, **p < 0.01.

Observations under a phase contrast microscope were conducted after 8 days of induction. ADSCs induced by the classical Dezawa’s method (In-ADSCs) exhibited significant morphological changes, showing a refractive pyramidal cell body with fine, bipolar or multiple processes (Figure 3A). ADSCs induced with RSC96-Exos (Exo-ADSCs) had spindle-like and small cell bodies and presented more elongated processes and stronger refraction than In-ADSCs (Figure 3A). To further verify the correlation between morphological changes and phenotypic changes in the gene and protein expression levels of SC markers, RT-qPCR, Western blot and immunofluorescence staining were performed on the cells induced for 8 days. The mRNA expression levels of S100ß, NGFR, MPZ, and GFAP were significantly upregulated in In-ADSCs and Exo-ADSCs compared with ADSCs (*p* < 0.01 for each) (Figure 3B). Exo-ADSCs exhibited significantly upregulated mRNA expression of NGFR (*p* < 0.05) compared with In-ADSCs, whereas there were no significant differences in the expression of the other indicators (Figure 3B). Western blot results further demonstrated that the protein levels of Schwann cell markers, namely S100ß, NGFR, MPZ, and GFAP, were significantly increased in both In-ADSCs and Exo-ADSCs compared with ADSCs, but no significant difference existed between In-ADSCs and Exo-ADSCs (Figure 3C). In addition, the results of immunofluorescence staining revealed that S100ß, NGFR, MPZ and GFAP were positively expressed in both In-ADSCs and Exo-ADSCs, while the expression of ADSCs was weakly positive, which was consistent with the RT-qPCR and Western blot results (Figure 3D). Taken together, the above data suggested that RSC96-Exos had a similar induction effect of ADSCs to Dezawa’s method.

### MiRNA Expression Profile of Exo-ADSCs

**FIGURE 4:**
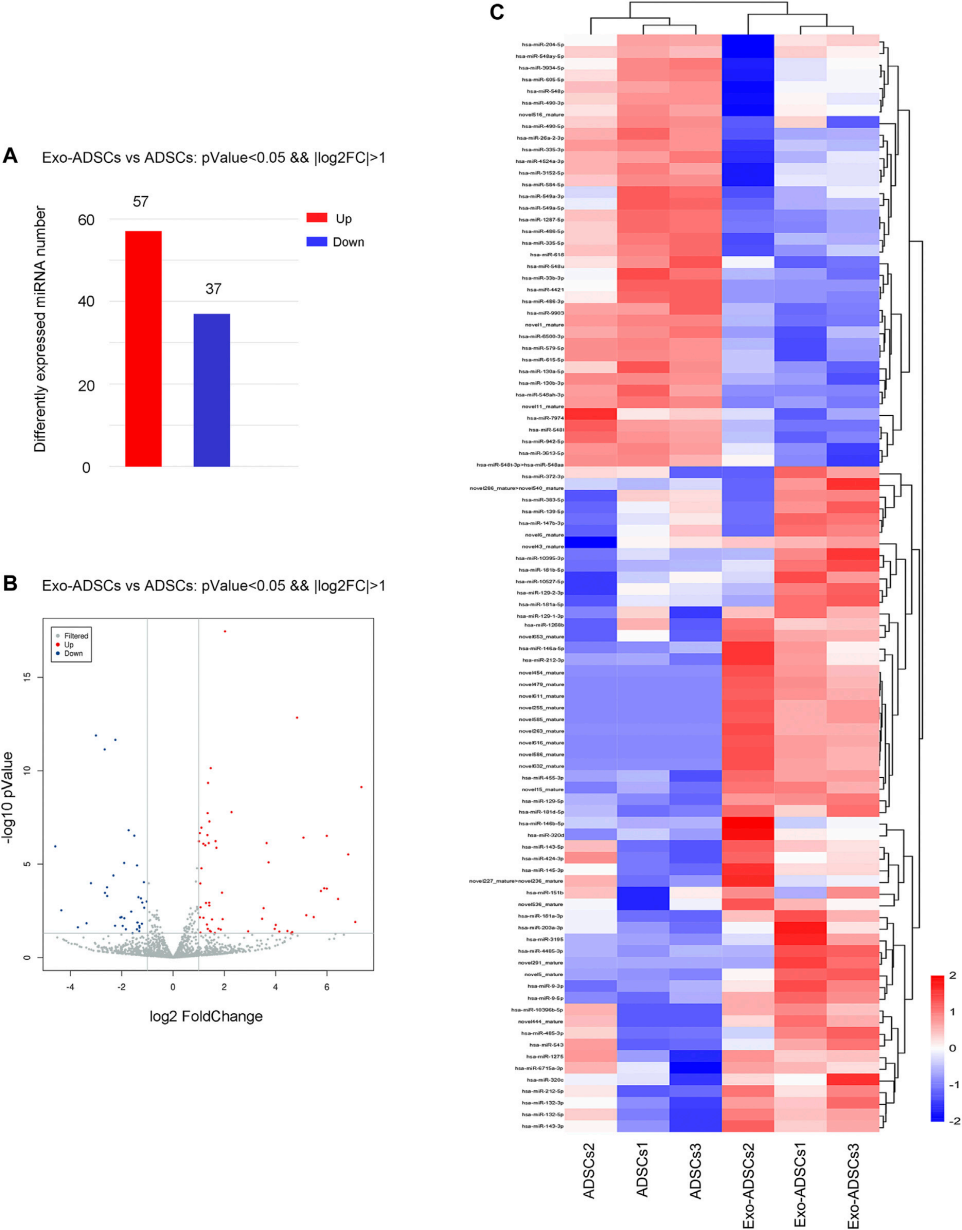
The miRNA expression profile of Exo-ADSCs. **(A)** The number of aberrantly expressed miRNAs with up- or downregulation. **(B)** Volcano map showing the distribution of differentially expressed miRNAs. Red dots, significantly upregulated genes. Blue dots, significantly downregulated genes. Gray dots, nondifferentially expressed genes. **(C)** Heat map showing the dominant cluster pattern of miRNAs. Red signals and blue signals represent upregulated and downregulated expression, respectively.

MiRNA sequencing was utilized to detect differentially expressed miRNAs in Exo-ADSCs and uninduced ADSCs. The sequencing results identified 94 miRNAs (Supplementary Table S3) with significantly different expression under the condition of “*p* < 0.05 and fold-change ≥ 2 or ≤0.5,” of which 72 were known miRNAs and 22 were novel miRNAs. Specifically, 57 miRNAs were upregulated and 37 miRNAs were downregulated (Figure 4A). The distribution of the differentially expressed miRNAs was shown in the form of a volcano plot (Figure 4B). A heat map (Figure 4C) demonstrated the dominant cluster pattern of these miRNAs, in which most of the differentially expressed miRNAs are associated with the cell regulation and developmental process, and may participate in the ADSCs’ differentiation process into Schwann cells.

### MiRNA Target Gene Prediction and GO Analysis

**FIGURE 5:**
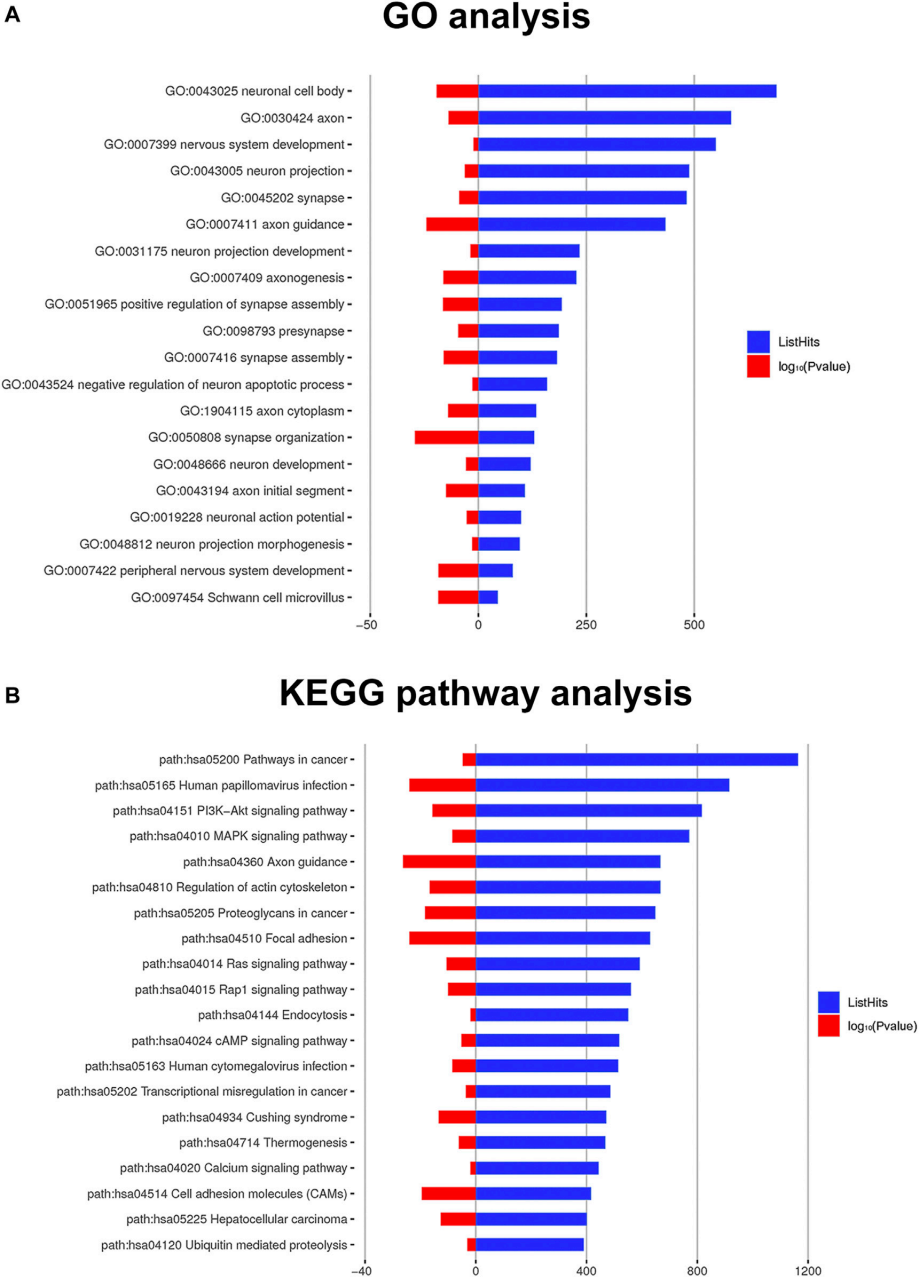
Significant GO terms and pathways of the target genes. **(A)** Twenty GO terms related to Schwann cells, neurons and axons are listed. **(B)** The top 20 significant KEGG pathway terms sorted by the number of genes (ListHits). The blue column represents the ListHits, while the red column represents the *p* value (-log10).

Through the miRanda database, a total of 7,636 target genes were predicted for the differentially expressed miRNAs. Subsequently, the biological processes, cellular components, and molecular functions of predicted target genes were analyzed through the GO database. A total of 3506 GO terms under conditions of ListHits >3 and *p* < 0.05 were enriched. Three types of significantly enriched GO terms attracted our attention: 1) GO terms related to Schwann cell or myelin function; 2) GO terms associated with neuronal or axonal function; and 3) GO terms linked to synaptic function. As shown in Figure 5A, the top 20 significantly enriched GO terms correlated with the abovementioned types were sorted by the number of gene enrichments (ListHits), and full details are shown in Supplementary Table S4. The target genes involved in these GO terms may play an important role in the differentiation of ADSCs into Schwann cells induced by RSC96-Exos.

### KEGG Pathway Analysis

Target genes were found to be enriched in KEGG signaling pathways. Under conditions of ListHits >3 and *p* < 0.05, a total of 98 significantly enriched KEGG terms were enriched. We ranked the top 20 terms (Figure 5B) from large to small according to the number of target genes enriched in each term (ListHits), which included the PI3K-Akt signaling pathway, MAPK signaling pathway, Ras signaling pathway and Rap1, and full details are shown in Supplementary Table S5. These signaling pathways may be involved in the process of exosome-induced differentiation into Schwann cells.

### MiRNA and Target Gene Screening and RT-qPCR Validation

**FIGURE 6:**
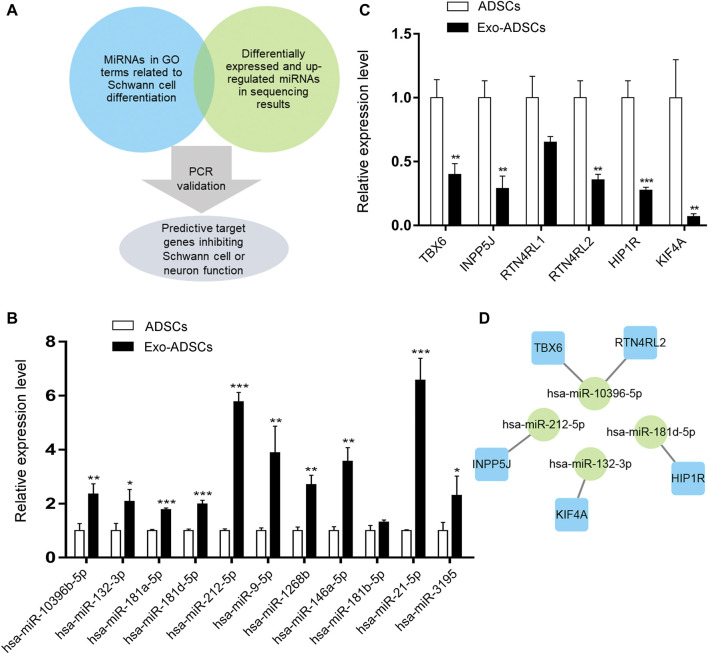
MiRNAs and target genes validation. **(A)** Mind map for miRNAs and target genes screening. **(B)** RT-qPCR analysis to validate the expression of the selected miRNAs in ADSCs and Exo-ADSCs. **(C)** RT-qPCR analysis to validate the expression of the selected target genes in ADSCs and Exo-ADSCs. **(D)** The miRNA-gene-network based on the validated miRNAs and their target mRNAs. Green circular nodes represent the validated miRNAs, whose target mRNAs were validated by RT-qPCR. Blue square nodes represent their validated target mRNAs. The results were presented as the mean ± SD (*n* = 3 independent experiments). *p < 0.05, **p < 0.01, ***p < 0.001.

When validating the sequencing results, we selected miRNAs whose target genes were enriched in GO terms associated with Schwann cells and neurons (Figure 5A). In addition, because exosomes contain miRNAs that are highly conserved among different species, the possible induction mechanism of ADSCs in our assumption is that rat exosomes may mediate the transfer of miRNAs from Schwann cells to human ADSCs as carriers, so miRNAs with upregulated expression in the sequencing result were selected. Eventually, we selected 11 miRNAs that matched the above conditions for RT-qPCR validation, and the validation results showed that hsa-miR-10396b-5p, hsa-miR-132-3p, hsa-miR-181a-5p, hsa-miR-181d-5p, hsa-miR-212-5p, hsa-miR-9-5p, hsa-miR-1268b, hsa-miR-146a-5p, hsa-miR-21-5p, and hsa-miR-3195 were significantly upregulated in Exo-ADSCs compared with those of ADSCs (*p* < 0.05) (Figure 6B), which was consistent with the sequencing results.

Since the effect of miRNAs on target genes is to inhibit transcription or degrade target mRNAs, when the selected miRNAs are upregulated, the expression levels of target genes are reduced. Therefore, in the validation of target genes, we selected target mRNAs that belonged to the validated miRNAs with downregulated expression in the sequencing results and were also reported to repress Schwann cell or neuronal function in the literature. These downregulated target mRNAs may thus promote ADSCs differentiation into Schwann cells. Figure 6A shows the mind map of screening miRNAs and target genes. Target gene validation results showed that TBX6, INPP5J, RTN4RL2, HIP1R and KIF4A were downregulated in Exo-ADSCs with significant differences (*p* < 0.01) (Figure 6C), which provided a basis for further exploration of the molecular mechanisms. Figure 6D shows the correspondence between the validated miRNAs and their predicted target genes.

### Validation of PI3K-Akt Signaling Pathway

**FIGURE 7:**
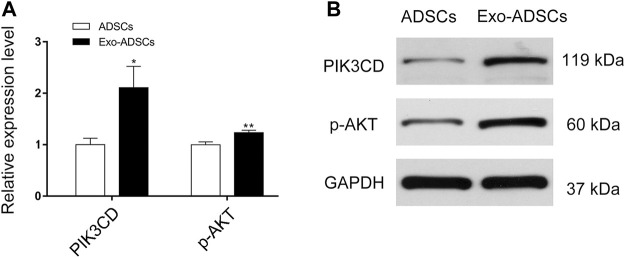
Validation of the PI3K-Akt signaling pathway. **(A)** RT-qPCR analysis of PIK3CD and Akt mRNA expression in ADSCs and Exo-ADSCs. **(B)** Western blot analysis of the protein levels of PIK3CD and phosphorylated Akt (p-Akt) in ADSCs and Exo-ADSCs. The data was presented as the mean ± SD (*n* = 3 independent experiments). *p < 0.05, **p < 0.01.

According to the KEGG pathway analysis, the PI3K-Akt signaling pathway was highly significant and enriched with a large number of target genes (Figure 5B), thus suggesting that the PI3K-Akt signaling pathway may play an important role in the differentiation of ADSCs into Schwann cells induced by exosomes. Therefore, we validated the PI3K-Akt signaling pathway by RT-qPCR and Western blot. The validation results showed that the mRNA expression level of PIK3CD, an isoform of PI3K, increased 2.1-fold in Exo-ADSCs of the experimental group and was significantly different (*p* < 0.05) (Figure 7A). Western blot analysis further verified that protein level of PIK3CD was significantly increased in Exo-ADSCs (Figure 7B). Although Akt, a downstream molecule in PI3K-Akt signaling, had only a 1.2-fold increase in mRNA transcript levels in Exo-ADSCs (*p* < 0.01) (Figure 7A), Western blot experiments showed that protein level of active phosphorylated Akt (p-Akt) was significantly increased (Figure 7B). This indicated that the PI3K-Akt signaling pathway was activated and involved in the process of RSC96-Exos-induced differentiation of ADSCs, specifically through the PIK3CD/Akt pathway.

## Discussion

Our study showed that Schwann cell-derived exosomes could induce human ADSCs to differentiate into Schwann cells *in vitro*. After induction, ADSCs expressed surface markers at different developmental stages of Schwann cells, including S100ß, NGFR, MPZ, and GFAP. Among them, S100ß and NGFR are the most commonly used markers to identify the differentiation of stem cells into Schwann cells (Dezawa et al., 2001; Juan et al., 2020). S100ß is not only expressed in Schwann progenitor cells at the early stage of Schwann cell development, but it is significantly expressed at all subsequent stages (Muppirala et al., 2020); NGFR plays an important role in Schwann cell myelination and cytokine secretion (Cosgaya et al., 2002); in addition, MPZ is the most important structural protein in peripheral nerve myelin (Raasakka et al., 2019). After acting on ADSCs, Schwann cell-derived exosomes promoted their expression of SC markers, and their expression levels were close to those induced by the classical Dezawa’s method, indicating that Schwann cell-derived exosomes could induce the differentiation of ADSCs into Schwann cells.

It has also been demonstrated that when undifferentiated mesenchymal stem cells are transplanted into rats, they can differentiate into corresponding Schwann cells after transplantation (Cuevas et al., 2002; Tohill et al., 2004; Chen et al., 2006), thus repairing injured nerves. This indicates that during nerve regeneration, there is a mechanism *in vivo* that can induce the differentiation of transplanted stem cells into Schwann cells, but which is still unknown. The previous hypothesis that some transplanted stem cells were induced to differentiate into Schwann cells by cytokines *in vivo* is mainly based on induction experiments *in vitro* using Dezawa’s method. However, it cannot be ignored that exosomes, as an important paracrine substance that contains various enzymes, RNAs and lipids, are more complex to construct. They may also be able to induce stem cell differentiation. Previous studies have reported that dedifferentiated Schwann cells are able to secrete exosomes to the injury site of sciatic nerves in rats *in vivo* (Lopez-Verrilli et al., 2013; López-Leal et al., 2020). Our experiment proved that human ADSCs could be induced to differentiate into Schwann cells by Schwann cell-derived exosomes, which provided a new possibility for the differentiation of transplanted stem cells into Schwann cells *in vivo*. Moreover, when the induction effect of Schwann cell-derived exosomes was confirmed, they may also be involved in the regulation process of Schwann cells themselves from dedifferentiation to redifferentiation during nerve regeneration, forming a feedback regulation loop, which needs to be verified by further experiments.

Although the directly transplanted stem cells would partially differentiate into Schwann cells *in vivo*, this transdifferentiation rate is relatively low, which has been reported by a literature that only 5% injected BMSCs with BrdU label expressed the Schwann cell marker (S100 immunoreactivity) *in vivo* (Cuevas et al., 2002). In contrast, our exosome-induction method allows a vast majority of ADSCs to differentiate into Schwann cell-like cells (SCLCs) *in vitro*, thus guarantying that the differentiated SCLCs could induce and speed up nerve repair and regeneration. SCLCs have noticeable advantages in promoting peripheral nerve regeneration compared with stem cells. The differentiated SCLCs could obviously promote neurite outgrowth and elongation by forming myelin around neurites, which has not been verified for undifferentiated stem cells (Dezawa et al., 2001). Additionally, SCLCs could exert a prominent paracrine effect to regenerate neuronal functions *via* producing higher amounts of several neurotrophic factors than that of stem cells, such as BDNF, NGF and GDNF (Liu et al., 2016). Consequently, our method *via* exosome induction is obviously superior to ADSCs transplantation alone in terms of the transdifferentiation rate, neurite regeneration, myelin formation, neurotrophic factors secretion, etc.

In previous studies, to obtain SCLCs from stem cells, the classical Dezawa’s method or other modified methods was mainly used *in vitro* (Dezawa et al., 2001; Liu et al., 2016; Saulite et al., 2018), including various types of neurotrophic factors and chemical reagents. These studies have proven that induced SCLCs are able to be transplanted into the site of nerve injury in rats *in vivo*, thus promoting the regeneration of injured nerves (Liu et al., 2016; Saulite et al., 2018). However, their clinical application is limited due to the toxic effects of chemical reagents (e.g., β-ME and forskolin) (White et al., 1973; Mohammed et al., 2015). In this study, we successfully used Schwann cell-derived exosomes to induce human ADSCs to differentiate into Schwann cells. Because exosomes are not immunogenic and have no risk of tumor formation (Adamiak et al., 2018), they are relatively safe for application.

The mechanism by which exosomes induce ADSCs to differentiate into Schwann cells is unclear. Because human-derived Schwann cells are difficult to extract, we used the rat Schwann cell line RSC96 for replacement. Although the inducing exosomes are not the same species as human cells, we successfully induced the differentiation of human ADSCs. Therefore, we speculated that a certain substance in rat exosomes can also act on human cells. Exosomes are perceived as mediators of intercellular communication, releasing the inner content of DNA, RNA, proteins, and lipids to cells or activating downstream signaling pathways through receptor-ligand interactions (Shao et al., 2018). The miRNAs in exosomes are highly conserved among species (Lee et al., 2007), so we suggested that miRNAs play an important role in inducing ADSC differentiation. To further explore the role of miRNAs, we conducted miRNA sequencing of Exo-ADSCs and subsequent bioinformatics analysis.

Through GO analysis, we detected 3,506 significantly enriched GO terms. In particular, the terms under biological process annotation mainly included nervous system development (GO: 0007399), axon guidance (GO: 0007411), neuron projection development (GO: 0031175), positive regulation of peripheral synapse assembly (GO: 0051965), neuron development (GO: 0048666), and nervous system development (GO: 0007422). Biological functions are mainly linked to the peripheral nervous system and neuronal differentiation and regulation. Furthermore, the terms under the cellular component annotation mainly included neuronal cell body (GO: 0043025), axon (GO: 0030424), and Schwann cell microvillus (GO: 0097454), which were associated with Schwann cells, neurons, and axons. The results of GO analysis are consistent with the differentiation of ADSCs into Schwann cells and suggest that differentiated cells may also have a role in regulating the peripheral nervous system, such as axons and neurons, which needs further verification.

Among the validated significantly upregulated miRNAs, the previous literature reported that the expression of miR-132 significantly increased during fetal rat brain development and differentiation of rat embryonic neural stem cells *in vitro* and increased synaptic proteins in differentiated cortical neurons (Yoshimura et al., 2016); miR-181d-5p could promote neurite growth *via* the PI3K/Akt signaling pathway (Shen et al., 2017); miR-212-5p attenuated ferroptotic neuronal death after traumatic brain injury by targeting Ptgs2 (Xiao et al., 2019); overexpression of miR-9-5p could inhibit the LPS-induced inflammatory response in myelinating oligodendrocytes (Yue et al., 2019); miR-146a-5p transported by nanoparticles could increase the expression of myelin basic protein and reduce inflammatory and demyelinating responses in diabetic peripheral neuropathy (Luo et al., 2019); and miR-21-5p was enriched in neuron-derived exosomes and could reduce Rab11a-mediated neuronal autophagy after traumatic brain injury (Li et al., 2019). It can be concluded that these differentially upregulated miRNAs have promoting or protective effects on the nervous system, but to our knowledge, few studies have reported their relationship with ADSCs differentiation into Schwann cells, which was demonstrated in our research.

We further focused on the target genes of these validated miRNAs and selected target genes with negative regulatory effects on the nervous system reported in the literature for validation. It was shown that knockdown of the transcription factor Tbx6 enhanced retinoic acid-induced differentiation of P19CL6 cells into neurons and glial cells and accelerated the rate of neurite formation (Gavrilov et al., 2012); INPP5J was involved in the control of glioblastoma cell migration (Ramos et al., 2018); the Nogo-66 receptors RTN4RL2 were binding proteins for many myelin-associated inhibitory factors (McDonald et al., 2011); HIP1R could bind to cytotoxic huntingtin-interacting protein I and influence its function (Chopra et al., 2000); kinesin KIF4A was present in all processes of immature cortical neurons, and its overexpression caused neuronal apoptosis (Heintz et al., 2014). After validation, the expression of the above target genes was downregulated, but the seed region of its respective miRNA as well as the precise mechanism of the induction effect on ADSCs need to be further confirmed by subsequent experiments.

To gain more insight into the signaling pathways in which target genes are involved, we performed KEGG pathway analysis. Among the significantly different signaling pathways, we ranked the number of enriched target genes (Listhits) in each term, and the signaling pathways with higher Listhits were PI3K-Akt signaling pathway, MAPK signaling pathway, Ras signaling pathway and Rap1 signaling pathway. Specifically, the PI3K-Akt signaling pathway is tightly associated with myelination. It has been reported in the literature that when Schwann cells were cocultured with DRG neurons *in vitro*, inhibition of PI3 kinase blocked Schwann cell elongation and subsequent myelination (Maurel and Salzer, 2000). Moreover, a study demonstrated that the impact of Akt overexpression in oligodendrocytes was enhanced myelination, and overexpression of Akt in Schwann cells did not impact myelination (Flores et al., 2008). This controversial finding led to further research. Elevated levels of phosphatidylinositol 3,4,5-trisphosphate (PIP3), a core mediator in the PI3K-Akt signaling pathway, have developmental effects on both oligodendrocytes and Schwann cells (Goebbels et al., 2010). An *in vivo* conducted study showed more findings that when sciatic nerve segments were transferred with Ax-myrAkt, they showed more myelinated axons with even distribution (Ogata et al., 2004). In our study, it was verified that both PIK3CD and p-Akt were upregulated, which may be associated with the process by which ADSCs differentiate into Schwann cells.

In terms of clinical translation, preinduction of stem cells into Schwann cells before *in vivo* transplantation is still too complicated. Since miRNAs are easy to synthesize *in vitro* and have a strong ability to regulate gene expression (Bernardo et al., 2015), the use of tissue engineering techniques such as nanoparticle liposomes to carry miRNAs for *in vivo* injection could be the next step in clinical translation (Lee et al., 2019). In a previous study on diabetic peripheral neuropathy (DPN), Luo et al. used nanoparticles to carry mimics of miR-146a-5p, which was then injected *in vivo* into the sciatic nerve lesion of rats, and found that nano-miR-146a-5p improved nerve conduction velocity and alle*via*ted the morphological damage and demyelination of the sciatic nerve of DPN rats, confirming the therapeutic effect of nanoparticles carrying miR-146a-5p (Luo et al., 2019). In a follow-up study, we will further investigate the biological function of the screened miRNAs and attempt to combine them with nanoparticles for *in vivo* injection into peripheral nerve injury sites for the treatment of peripheral nerve injury.

## Conclusion

In this study, we demonstrated that Schwann cell-derived exosomes could induce ADSCs to differentiate into Schwann cells *in vitro* and reported the miRNA expression profile of Schwann-like ADSCs after differentiation. Furthermore, the prediction of the potential function and mechanism of differential miRNAs and their target genes by bioinformatics analysis was performed, following verification of their expression levels by RT-qPCR and the activation of the PI3K-Akt signaling pathway during ADSC differentiation. These results provide a new possibility for stem cell therapy of peripheral nerve injury, which may improve the treatment effect by transplanting miRNA vectors in the future, thereby enriching the therapeutic means of peripheral nerve injury.

## Data Availability

The datasets presented in this study can be found in online repositories. The names of the repository/repositories and accession number(s) can be found below: https://www.ncbi.nlm.nih.gov/geo/, GSE183623.
