# Supplementary material for: Schwann Cell-Derived Exosomes Induce the Differentiation of Human Adipose-Derived Stem Cells Into Schwann Cells
Source: Front Mol Biosci. 2022 Jan 31;8:835135. doi: 10.3389/fmolb.2021.835135 (PMC8841477; doi:10.3389/fmolb.2021.835135)
Supplement: Supplementary file 2 [file Table1.DOCX]

**Table S1.** MRNAs’ primers used for real-time polymerase chain reaction

| Gene | Primer sequences (5′–3′) |
| --- | --- |
| S100ß  NGFR  MPZ  GFAP  RTN4RL1  RTN4RL2  KIF4A  HIP1R  INPP5J  PIK3CD  AKT  GAPDH | F: AGGAAGAGGATGTCTGAGCTGG  R: AGTCACATTCGCCGTCTCCAT  F: CACCGACAACCTCATCCCTGT  R: GCTGTTGGCTCCTTGCTTGTT  F: AGGCTCAGTGCTATGGAGAAGG  R: GCCCGCTAACCGCTATTTCTT  F: AGGTCCATGTGGAGCTTGAC  R: GCCATTGCCTCATACTGCGT  F: CCGCAATCAGATCTCTAAGG  R: GGCATGATGTCAAAACTGAAC  F: GCTGGACCTCGGTGACAA  R: CTGGCAGCGGTACAAATG  F: TCTGCCATGTTGTTGAAGC  R: GAAGGATCTTCCCAAACAGTAG  F: GAAGAGAGAGGTGGAAATGC  R: TCTTCAGCTGCGCGATGTA  F: GGATCGGCTTATACCGGGTG  R: GAAGGCAGCGAGATCTGGAA  F: GCTCTGCCAGTTCCAGTACA  R: TTGCTCTGCTCATCCCGCAT  F: CTGCACAAACGAGGGGAGT  R: GCGCCACAGAGAAGTTGTTG  F: AAAATCAAGTGGGGCGATGCT  R: TGGTTCACACCCATGACGAAC |
