# Supplementary material for: Schwann Cell-Derived Exosomes Induce the Differentiation of Human Adipose-Derived Stem Cells Into Schwann Cells
Source: Front Mol Biosci. 2022 Jan 31;8:835135. doi: 10.3389/fmolb.2021.835135 (PMC8841477; doi:10.3389/fmolb.2021.835135)
Supplement: Supplementary file 3 [file Table2.DOCX]

**Table S2.** MiRNAs’ primers used for real-time polymerase chain reaction

| Gene | Primer sequences (5′–3′) |
| --- | --- |
| hsa-miR-10396b-5p  hsa-miR-132-3p  hsa-miR-181a-5p  hsa-miR-181d-5p  hsa-miR-212-5p  hsa-miR-9-5p  hsa-miR-1268b  hsa-miR-146a-5p  hsa-miR-181b-5p  hsa-miR-21-5p  hsa-miR-3195  U6 | AAAACGGCGGGGCTCGGAG  CAGTCTACAGCCATGGTCG  AACATTCAACGCTGTCGGTG  AACATTCATTGTTGTCGGTGG  CTTGGCTCTAGACTGCTTACT  TCTTTGGTTATCTAGCTGTATGA  GTGGTGGTGGGGGTGAAA  GCAGTGAGAACTGAATTCCA  AACATTCATTGCTGTCGGTGG  TAGCTTATCAGACTGATGTTGA  CGCCGGGCCCGGGTT  F: GGAACGATACAGAGAAGATTAGC  R: TGGAACGCTTCACGAATTTGCG |
