# Supplementary material for: Schwann Cell-Derived Exosomes Induce the Differentiation of Human Adipose-Derived Stem Cells Into Schwann Cells
Source: Front Mol Biosci. 2022 Jan 31;8:835135. doi: 10.3389/fmolb.2021.835135 (PMC8841477; doi:10.3389/fmolb.2021.835135)
Supplement: Supplementary file 4 [file Table5.DOCX]

**Table S5.** Selected KEGG terms

| Term_ID | Term_description | ListHits | pValue |
| --- | --- | --- | --- |
| path:hsa05200 | Pathways in cancer | 1164 | 1.98E-05 |
| path:hsa05165 | Human papillomavirus infection | 916 | 1.14E-24 |
| path:hsa04151 | PI3K-Akt signaling pathway | 816 | 2.74E-16 |
| path:hsa04010 | MAPK signaling pathway | 771 | 3.75E-09 |
| path:hsa04360 | Axon guidance | 667 | 5.46E-27 |
| path:hsa04810 | Regulation of actin cytoskeleton | 667 | 2.54E-17 |
| path:hsa05205 | Proteoglycans in cancer | 649 | 4.60E-19 |
| path:hsa04510 | Focal adhesion | 630 | 1.04E-24 |
| path:hsa04014 | Ras signaling pathway | 592 | 3.06E-11 |
| path:hsa04015 | Rap1 signaling pathway | 560 | 1.15E-10 |
| path:hsa04144 | Endocytosis | 551 | 0.012926624 |
| path:hsa04024 | cAMP signaling pathway | 518 | 7.31E-06 |
| path:hsa05163 | Human cytomegalovirus infection | 514 | 3.83E-09 |
| path:hsa05202 | Transcriptional misregulation in cancer | 486 | 0.000249422 |
| path:hsa04934 | Cushing syndrome | 471 | 3.83E-14 |
| path:hsa04714 | Thermogenesis | 468 | 8.34E-07 |
| path:hsa04020 | Calcium signaling pathway | 444 | 0.0113827 |
| path:hsa04514 | Cell adhesion molecules (CAMs) | 416 | 2.93E-20 |
| path:hsa05225 | Hepatocellular carcinoma | 401 | 2.51E-13 |
| path:hsa04120 | Ubiquitin mediated proteolysis | 390 | 0.000957016 |
