# Supplementary material for: Schwann Cell-Derived Exosomes Induce the Differentiation of Human Adipose-Derived Stem Cells Into Schwann Cells
Source: Front Mol Biosci. 2022 Jan 31;8:835135. doi: 10.3389/fmolb.2021.835135 (PMC8841477; doi:10.3389/fmolb.2021.835135)
Supplement: Supplementary file 5 [file Table4.DOCX]

| Term_ID | Term_description | ListHits | pValue |
| --- | --- | --- | --- |
| GO:0043025 | neuronal cell body | 690 | 4.83E-20 |
| GO:0030424 | axon | 585 | 1.94E-14 |
| GO:0007399 | nervous system development | 550 | 0.005831681 |
| GO:0043005 | neuron projection | 488 | 5.76E-07 |
| GO:0045202 | synapse | 482 | 1.27E-09 |
| GO:0007411 | axon guidance | 433 | 1.10E-24 |
| GO:0031175 | neuron projection development | 234 | 0.000243726 |
| GO:0007409 | axonogenesis | 227 | 6.92E-17 |
| GO:0051965 | positive regulation of synapse assembly | 193 | 4.39E-17 |
| GO:0098793 | presynapse | 186 | 5.57E-10 |
| GO:0007416 | synapse assembly | 182 | 7.96E-17 |
| GO:0043524 | negative regulation of neuron apoptotic process | 159 | 0.002240153 |
| GO:1904115 | axon cytoplasm | 134 | 1.08E-14 |
| GO:0050808 | synapse organization | 129 | 4.71E-30 |
| GO:0048666 | neuron development | 121 | 1.77E-06 |
| GO:0043194 | axon initial segment | 108 | 1.21E-15 |
| GO:0019228 | neuronal action potential | 99 | 3.61E-06 |
| GO:0048812 | neuron projection morphogenesis | 96 | 0.001461044 |
| GO:0007422 | peripheral nervous system development | 80 | 4.13E-19 |
| GO:0097454 | Schwann cell microvillus | 45 | 2.62E-19 |

**Table S4.** Selected Go terms
